# Cost–Benefit Analysis of the COPE Program for Persons Living With Dementia: Toward a Payment Model

**DOI:** 10.1093/geroni/igab042

**Published:** 2021-10-16

**Authors:** Laura T Pizzi, Eric Jutkowitz, Katherine M Prioli, Ember (Yiwei) Lu, Zachary Babcock, Heather McAbee-Sevick, Dorothy B Wakefield, Julie Robison, Sheila Molony, Catherine V Piersol, Laura N Gitlin, Richard H Fortinsky

**Affiliations:** 1 Center for Health Outcomes, Policy & Economics, Rutgers University, Piscataway, New Jersey, USA; 2 Department of Health Services, Policy & Practice, Brown University School of Public Health, Providence, Rhode Island, USA; 3 Center of Innovation in Long Term Services and Supports, Providence VA Medical Center, Providence, Rhode Island, USA; 4 Ernest Mario School of Pharmacy, Rutgers University, Piscataway, New Jersey, USA; 5 Center on Aging, University of Connecticut, Farmington, Connecticut, USA; 6 School of Nursing, Quinnipiac University, Hamden, Connecticut, USA; 7 Department of Occupational Therapy, Thomas Jefferson University, Philadelphia, Pennsylvania, USA; 8 College of Nursing and Health Professions and AgeWell Collaboratory, Drexel University, Philadelphia, Pennsylvania, USA

**Keywords:** Health care policy, Health economics, Home- and community-based services, Medicaid/Medicare, Pragmatic trial

## Abstract

**Background and Objectives:**

There is a critical need for effective interventions to support quality of life for persons living with dementia and their caregivers. Growing evidence supports nonpharmacologic programs that provide care management, disease education, skills training, and support. This cost–benefit analysis examined whether the Care of Persons with Dementia in their Environments (COPE) program achieves cost savings when incorporated into Connecticut’s home- and community-based services (HCBS), which are state- and Medicaid-funded.

**Research Design and Methods:**

Findings are based on a pragmatic trial where persons living with dementia and their caregiver dyads were randomly assigned to COPE with HCBS, or HCBS alone. Cost measures included those relevant to HCBS decision makers: intervention delivery, health care utilization, caregiver time, formal care, and social services. Data sources included care management records and caregiver report.

**Results:**

Per-dyad mean cost savings at 12 months were $2 354 for those who received COPE with a mean difference-in-difference of −$6 667 versus HCBS alone (95% CI: −$15 473, $2 734; not statistically significant). COPE costs would consume 5.6%–11.3% of Connecticut’s HCBS annual spending limit, and HCBS cost-sharing requirements align with participants’ willingness to pay for COPE.

**Discussion and Implications:**

COPE represents a potentially cost-saving dementia care service that could be financed through existing Connecticut HCBS. HCBS programs represent an important, sustainable payment model for delivering nonpharmacological dementia interventions such as COPE.

**Translational Significance:** This study presents a robust economic analysis of a dementia support program, Care of Persons with Dementia in their Environments (COPE), tested in a pragmatic trial as a service embedded in the Connecticut home- and community-based services (HCBS) program. The COPE program resulted in net cost savings in several direct health care cost categories and can be delivered through the existing Connecticut HCBS program. We also present a potential payment model for this program which could be adapted to other states’ HCBS programs.

## Background and Objectives

In the United States, 5.8 million people are living with Alzheimer’s disease and related dementias (ADRDs), and two-thirds of $290 billion annual dementia-related health care costs are borne by Medicare and Medicaid (1). Added to this cost is the time value of caregiving provided by family/friends to persons living with dementia (2). Due to the lack of interventions to slow, halt, or reverse dementia’s pathology coupled with the aging of America, both direct and indirect dementia-related costs are expected to increase in the foreseeable future.

Given these trends, there is a critical need for effective interventions to support quality of life for persons living with dementia and their caregivers. Growing evidence supports nonpharmacologic programs that provide care management (CM), disease education, skills training, and support (3–5). Most evidence to date has been derived from randomized control trials, which may not be reflective of everyday life, representing a need to further test their effectiveness in a real-world home- and community-based service (HCBS) setting (6–10).

This study presents a cost–benefit analysis (CBA) conducted alongside the Care of Persons with Dementia in their Environments (COPE) program in Connecticut (“COPE-CT”). COPE-CT is the first known pragmatic and randomized trial to determine the effectiveness of an evidence-based dementia care intervention into a state HCBS program (11). HCBS are offered in more than 40 states to support the care of the most vulnerable members of the population (12). The Connecticut HCBS program for older adults, formally known as the Connecticut Home Care Program for Elders (CHCPE), is state- and Medicaid-funded and provides different categories of benefits based on functional status and income (13). The CHCPE categories also have different monthly spending limits and participant cost-sharing requirements (13). CHCPE Categories 1 and 2 are state-funded and Category 3 is Medicaid waiver-funded. Categories 1 and 2 participants have 9% cost sharing on the total cost of benefits provided, while Category 3 has no cost-sharing requirements. Of the 16 000 CHCPE beneficiaries served annually, approximately 25%–30% have an ADRD diagnosis (11).

The main goal of our CBA was to determine if COPE delivered with HCBS resulted in cost savings compared to HCBS alone (usual care). Secondarily, we sought to examine a potential payment model whereby COPE is a covered service under Connecticut HCBS, based on its fit within monthly spending limits. Finally, we examined whether caregivers’ willingness to pay (WTP) for COPE falls within CHCPE cost-sharing levels.

## Research Design and Methods

### Description of the Pragmatic Trial

The COPE-CT pragmatic trial has been described elsewhere (11,14). Briefly, persons living with dementia and caregiver dyads were enrolled after eligibility screening and informed consent (14). To be eligible, the person living with dementia had to be enrolled in CHCPE and receive a monthly home care plan from Connecticut Community Care (CCC, a CM provider), have a diagnosis of dementia or ≥4 errors on the Mental Status Questionnaire (indicative of moderate cognitive impairment), and speak English. Caregivers had to be ≥21 years old, willing and able to participate in the study, plan to live in the area for 12 months, and speak English.

Consistent with a pragmatic approach, CCC was directly involved in planning and implementing the study. CCC is Connecticut’s largest CM organization supporting CHCPE (14). CCC’s care managers annually evaluate the person living with dementia’s needs and create personalized HCBS care plans. CCC care managers identified CHCPE clients and their caregiver dyads who were potential study candidates by examining their client lists and during routine phone calls, explaining study features to provisionally eligible clients and/or their caregivers. Interested dyads were referred to the research team at the University of Connecticut Center on Aging who conducted final screening and consent.

Dyads randomized to the COPE group received up to 10 in-home sessions delivered by an occupational therapist (OT), as well as 1 in-home visit and 1 telephone call by an advanced practice nurse (APN) (14). During the OT sessions, the person living with dementia was assessed for functional limitations and environmental stressors, and the OT worked with the caregiver to develop solutions for up to 3 key concerns identified by the caregiver. The OT also offered strategies to the caregiver to prevent falls in the home, which in persons living with dementia are a leading cause of hospitalizations (15), along with strategies to optimize physical activity and activity engagement. During the APN visit, the person living with dementia received a physical examination and routine blood and urine analyses. The APN also conducted a medication review as part of the home visit to check for polypharmacy-related concerns, explain any potential concerns to the caregiver, and discuss with the caregiver how to raise medication-related issues with the physician of the person living with dementia. Additionally, the APN provided education for the caregiver about how to check the person living with dementia for signs of dehydration, unexpressed pain, constipation, and signs of infections. The purpose of the APN component was to identify any underlying medical concerns that could contribute to functional challenges or behavioral and psychological symptoms. The APN communicated laboratory results during a follow-up telephone call to the caregiver (and, if requested by the caregiver, directly to the person living with dementia’s primary medical provider). The study was approved by the University of Connecticut institutional review board, inclusive of the trial and cost study reported herein.

Persons living with dementia and caregiver outcomes were assessed over 4 months by interviewers who remained masked to group allocation. Persons living with dementia outcomes included level of functional dependence, behavioral and psychological symptoms, engagement in activities, and quality of life (11). Caregiver outcomes included perceived well-being, self-confidence in using strategies to manage dementia, and level of distress due to the person living with dementia’s behavioral and psychological symptoms (11). Trial results showed caregivers reported better perceived well-being after 4 months of COPE compared to caregivers receiving usual care. After 12 months of COPE, the persons living with dementia were more engaged in meaningful activities compared to those receiving usual care (11).

### Cost Study Approach

We estimated the net cost of delivering COPE relative to HCBS usual care based on cost data obtained during the trial and assessed whether the net financial benefit of COPE was ≥$0. The cost analysis was conducted from a HCBS decision-maker perspective and included 130 dyads in the COPE plus HCBS group and 120 dyads in HCBS only for whom CCC data were available, representing 86% of the main study sample (consort chart in Supplementary Figure A1). Among dyads randomized to COPE, caregivers in the cost sample compared to the noncost sample were more likely to be female and daughters of the person living with dementia (*p* = .01). Because the noncost study sample for the COPE group was small (15 participants), these differences could be due to chance. There were no differences between participants in the cost study and those in the noncost study for the usual care group (Supplementary Table A1).

Costs were measured for both study groups and included direct and indirect costs. We measured the direct cost of delivering COPE or usual care (staff time costs, travel, and supplies), health care services, and formal care and social services. We also measured indirect costs as the dollar value of caregivers’ time spent actively caring for or supervising the person living with dementia. Although the value of caregiving is a societal cost and not a direct cost to HCBS funders (16), we included it in the analysis due to its relevance for dementia. We multiplied all time-based activity by applicable wage rates.

All costs are presented in $US 2019 (17). Costs from 1 year prior to randomization (“baseline”) were compared to the year postrandomization (“12 months”) using a difference-in-difference (DID) approach. Though the intervention is delivered over 4 months, we used a 1-year pre-/postperiod time horizon to increase the relevance of findings to HCBS decision makers.

### Delivery of Intervention or Usual Care Control Costs

We captured the COPE and usual care delivery cost using an investigator-developed template (18,19). Additional resources required for delivering COPE included staff training and supervision (consisting of case presentation debriefings with a seasoned OT to ensure treatment fidelity), OT/APN time with clients and preparing to meet with clients, travel time and mileage (reimbursement rate of $0.58/mile), and laboratory testing (20).

Costs for laboratory tests performed by the APN, activity supplies, and training materials were obtained from project accounting records.

Wage rates for all personnel were obtained from U.S. Bureau of Labor Statistics (BLS) occupational data (21) and were inflated by 31.3% to account for fringe benefits (22). Personnel time costs were then inflated from $US 2015 to $US 2019 (the most recent year of inflation data available) (17).

Univariate sensitivity analyses were conducted on COPE intervention costs to identify ways to deliver the program more efficiently. The cost of formal staff training was examined at $0, consistent with a scenario where interventionists were already trained to deliver COPE. Telephone calls outside of the COPE (eg, appointment reminder calls or connecting with other community resources) were omitted under the assumption that they could be converted to less costly and asynchronous forms of communication (eg, e-mails or text messages). Round-trip travel time was capped at 40 minutes and round-trip travel miles were capped to 20 miles, simulating a smaller geographic service area. The cost of interventionist debriefing was tested at half its base case cost, as would be the case if OTs were already proficient with COPE. Finally, the minimum cost to deliver COPE was calculated by varying all COPE program components to their low ends simultaneously.

### Health Care Utilization

Health care utilization was obtained from the CCC database (informed through monthly check-in calls with the person living with dementia and/or caregivers by the CCC care manager) or caregiver interviews (Supplementary Table A2) for the following categories: nursing home stays (including long-term and rehabilitation stays), respite care, inpatient hospitalizations, emergency department, outpatient visits, medications, durable medical equipment, nurse visits, and home health aide (HHA) visits. The battery used for capturing this information was developed based on validated survey instruments (23,24).

Nursing home length of stay was calculated from dates available in CCC data. Long-term stays were costed by multiplying the number of days in the nursing home by published daily rates for each facility (25). Rehabilitation stays were costed using the FY2015 unadjusted federal per-diem rate for urban facilities (26).

For inpatient hospitalizations and emergency department visits, International Classification of Disease-9/10 diagnosis codes in CCC data were used to estimate mean charges for that diagnosis per HCUPnet (27), downward adjusted to approximate health care costs assuming a cost-to-charge ratio of 1/3.4 (28).

Outpatient visits were recorded during the caregiver study assessments (visits to primary care physicians, geriatricians, neurologists, psychiatrists, psychologists, physical therapists, OTs, and speech language pathologists). For each visit type, costs were estimated by multiplying the number of visits by the 50th percentile current procedural terminology costs per the 2015 National Fee Analyzer (29), inflated to $US 2019 (Supplementary Table A3) (17).

Visiting nurse and HHA services were also captured from the caregiver study assessments and were assumed to be 30 minutes for visiting nurse and 1 hour for HHA.

Medication data in CCC included drug name, strength, dosage, frequency, and date(s) that the CCC care manager recorded the medication. For medication entries missing dosage, strength, or frequency, a seasoned pharmacist applied standard regimens based on product prescribing information (11). Costs were estimated by multiplying the number of doses by per-dose wholesale acquisition costs as found in the published databases (30,31). Durable medical equipment, recorded in the CCC data, was costed by applying reimbursement values (32).

Respite care stays were recorded in caregiver study assessments and were specified as overnight care. Based on the services that a respite care facility typically provides, a proxy cost for respite care days was calculated as 8 hours of HHA time per day spent in the respite care facility, plus fringe benefits (21,22).

### Formal Care and Social Services

Caregivers reported the person living with dementia’s visits from social workers, meals delivered, transportation (to reach any form of necessity such as medical visits, grocery shopping, etc.), and visits to an adult day care center. Social worker visits were costed by assuming each visit was 1 hour, and applying the mean Connecticut social worker wage rate in 2015 ($31.09/h) and fringe benefits rate, then inflating to $US 2019 (17,21,22). Meals were costed by applying a per-meal cost of $11.92 to the number of delivered meals received by the person living with dementia (33). Transportation costs were based on the senior bus fare of $1.50 per round trip in $US 2015 and inflated to $US 2019, applied to the number of round trips taken by the person living with dementia. Adult day care services were based on an hourly rate of $12.80 (in $US 2019), and assuming 8 h/day for each day of service reported (34).

### Caregiver Time Costs

Caregivers reported the weekly hours they spent supervising and assisting the person living with dementia with activities of daily living and instrumental activities of daily living. We capped caregiving time to 16 h/day based on references from published literature (35–37). If the caregiver was currently employed, their time was costed by mapping their reported occupation to standard BLS occupational wages plus fringe benefits in $US 2015, then inflating to $US 2019 (17,21,22). If the caregiver was not employed or if their occupation was missing, the time was costed assuming a HHA wage rate plus fringe benefits (17,21,22).

### Net Financial Benefit

For each cost type, mean per-dyad cost differences from baseline to 12 months were calculated by group assignment and were compared between groups via a series of 18 familywise Wilcoxon Rank-Sum tests using the Bonferroni-corrected significance threshold of α _adjusted_ = .05/18 = .00278.

### Assessment of Potential COPE Financing Through the Connecticut HCBS Program

To assess whether COPE’s intervention costs fit in the context of CHCPE spending limits, we calculated the percentage of total CHCPE available benefits that would be required to pay for COPE over 12 months, accounting for concurrent CM services and considering that the program itself would be delivered for only 4 months out of the benefit year. Care plan limits were obtained from the Connecticut Department of Social Services for the CHCPE categories (13). We conducted this assessment only for CHCPE Categories 2 and 3 because the trial revealed these categories were most common in the population of interest for COPE (over 99% of trial sample population). A flat 40% overhead was added to our COPE intervention costs to reflect recent data on general administrative costs for home care agencies (38). The goal of this analysis was to calculate the percentage of CHCPE care plan funds that would be required to pay for COPE over a 12-month benefit year, with the equation shown below:


$$\begin{array}{ll} & \%Utilized\;by\;COPE/year=\\ & \;\frac{Care\;plan\;limits/month\times 12\;months}{\left((COPE+overhead\;costs\right)\times 4\;months)+(CM\;costs/month\times 12\;months)} \end{array}$$

Note that CM refers to care management services outside of COPE, which would be necessary to continue in this population. A conceptual illustration of the payment model is provided in Figure A2.

### Assessment of Caregiver’s WTP for COPE in the Context of Connecticut HCBS Program Cost Sharing

We assessed caregivers’ WTP for COPE at 12 months using a contingent valuation method. The purpose of capturing WTP was to understand the monetary value that caregivers placed on COPE after receiving the program, which in turn is useful to consider potential cost-sharing amounts. WTP response options ranged from $0 to $200 per COPE session.

## Results

Demographics for the cost sample population are in Table 1. The majority of persons living with dementia were in CHCPE Categories 2 (*n* = 75; 30%) or 3 (*n* = 172; 68.8%). Caregivers and persons living with dementia mean ages were 62.5 (*SD* 10.9) and 85.2 years (*SD* 7.9), respectively. Both caregivers and persons living with dementia were predominantly female (71.6% of caregivers and 76.0% of persons living with dementia). A majority of persons living with dementia were Caucasian (*n* = 189; 75.6%), followed by Black (*n* = 46; 18.4%) and other ethnicities (*n* = 15; 6.0%).

**Table 1.** Cost Sample Demographics, Overall and by Group

Characteristic	Overall *N* = 250^*a*^	COPE *n* = 130^*a*^	Usual Care *n* = 120^*a*^	*p* Value^*b*^
CHCPE category				.957
1	3 (1.2%)	2 (1.5%)	1 (0.8%)	
2	75 (30.0%)	38 (29.2%)	37 (30.8%)	
3	172 (68.8%)	90 (69.2%)	82 (68.3%)	
Caregiver age, mean (*SD*)	62.5 (10.92)	62.1 (11.25)	63.0 (10.58)	.4889
Person living with dementia age, mean (*SD*)	85.2 (7.92)	85.1 (8.31)	85.4 (7.50)	.7412
Caregiver gender				**.0074**
Female	179 (71.6%)	103 (79.2%)	76 (63.3%)	
Male	71 (28.4%)	27 (20.8%)	44 (36.7%)	
Person living with dementia gender				.4597
Female	190 (76.0%)	96 (73.8%)	94 (78.3%)	
Male	60 (24.0%)	34 (26.2%)	26 (21.7%)	
Caregiver race				.4618
White, Caucasian	189 (75.6%)	95 (73.1%)	94 (78.3%)	
Black, African-American	46 (18.4%)	27 (20.8%)	19 (15.8%)	
Other	12 (4.8%)	6 (4.6%)	6 (5.0%)	
Native American or Alaska native	2 (0.8%)	2 (1.5%)	0 (0.0%)	
Unknown/no response	1 (0.4%)	0 (0.0%)	1 (0.8%)	
Person living with dementia race				.7543
White, Caucasian	192 (76.8%)	98 (75.4%)	94 (78.3%)	
Black, African-American	44 (17.6%)	25 (19.2%)	19 (15.8%)	
Other	13 (5.2%)	7 (5.4%)	6 (5.0%)	
Native American or Alaska native	1 (0.4%)	0 (0.0%)	1 (0.8%)	
Unknown/no response	0 (0.0%)	0 (0.0%)	0 (0.0%)	
Caregiver highest education attained				.1909
College/postgraduate	116 (46.4%)	66 (50.8%)	50 (41.7%)	
HS or less	72 (28.8%)	31 (23.8%)	41 (34.2%)	
Some college	61 (24.4%)	32 (24.6%)	29 (24.2%)	
Unknown/no response	1 (0.4%)	1 (0.8%)	0 (0.0%)	
Person living with dementia highest education attained				.6108
HS graduate	139 (55.6%)	73 (56.2%)	66 (55.0%)	
Less than HS	71 (28.4%)	34 (26.2%)	37 (30.8%)	
College/postgraduate	40 (16.0%)	23 (17.7%)	17 (14.2%)	
Caregiver employment status				.5996
Not working	120 (48.0%)	64 (49.2%)	56 (46.7%)	
Full time (>35 h/week)	99 (39.6%)	48 (36.9%)	51 (42.5%)	
Part-time (<35 h/week)	31 (12.4%)	18 (13.8%)	13 (10.8%)	
Caregiver difficulty paying for the basics				.3392
Not difficult at all	116 (46.4%)	67 (51.5%)	49 (40.8%)	
Somewhat difficult	67 (26.8%)	33 (25.4%)	34 (28.3%)	
Not very difficult	45 (18.0%)	20 (15.4%)	25 (20.8%)	
Very difficult	21 (8.4%)	9 (6.9%)	12 (10.0%)	
Unknown/no response	1 (0.4%)	1 (0.8%)	0 (0.0%)	
Caregiver marital status				.5830
Married or living as married	147 (58.8%)	74 (56.9%)	73 (60.8%)	
Divorced/separated	44 (17.6%)	24 (18.5%)	20 (16.7%)	
Never married	42 (16.8%)	25 (19.2%)	17 (14.2%)	
Widowed, not currently married	17 (6.8%)	7 (5.4%)	10 (8.3%)	
Caregiver relationship to person living with dementia				.1268
Daughter	140 (56.0%)	78 (60.0%)	62 (51.7%)	
Spouse	44 (17.6%)	23 (17.7%)	21 (17.5%)	
Son	40 (16.0%)	14 (10.8%)	26 (21.7%)	
Other	26 (10.4%)	15 (11.5%)	11 (9.2%)	
Caregiver and person living with dementia living arrangement				.8987
Live together	143 (57.2%)	75 (57.7%)	68 (56.7%)	
Live apart	107 (42.8%)	55 (42.3%)	52 (43.3%)	

*Notes*: CHCPE = Connecticut Home Care Program for Elders; COPE = Care of Persons with Dementia in their Environments; HS = high school.

^*a*^Statistics presented: mean (*SD*) or *n* (%).

^*b*^Statistical tests performed: two-sided *t* test and Fisher’s exact test. All tests were performed at the alpha = 0.05 level.

In examining costs, the total per-dyad mean cost differences at 12 months were −$2 354 for COPE and $4 313 for usual care, yielding a mean DID of −$6 667 (95% CI: −$15 473, $2 734), representing a cost saving for COPE (Table 2). Utilization of these services (without conversion to $USD) is provided in Supplementary Table A4.

**Table 2.** Mean per-Dyad Direct and Indirect Costs for the COPE Intervention vs Usual Care

Cost Type	COPE Intervention (*n* = 130)			Usual Care Control (*n* = 120)				
	BL	M12	M12–BL	BL	M12	M12–BL	DID (95% CI)	*p* Value^*a*^
*Direct costs: delivery of intervention or usual care control*								
Formal staff training	0.00	218.75	218.75	0.00	0.00	0.00	218.75	—
Screening for program eligibility	0.00	3.87	3.87	0.00	3.87	3.87	0.00	—
OT/APN time with client	0.00	677.48	677.48	0.00	0.00	0.00	677.48	—
OT/APN work outside of intervention	0.00	59.79	59.79	0.00	0.00	0.00	59.79	—
Travel time to participant homes	0.00	402.79	402.79	0.00	0.00	0.00	402.79	—
Mileage for travel to participant homes	0.00	170.63	170.63	0.00	0.00	0.00	170.63	—
Interventionist debriefing	0.00	177.43	177.43	0.00	0.00	0.00	177.43	—
Activity supplies and assessment materials	0.00	60.34	60.34	0.00	0.14	0.14	60.21	—
Laboratory testing	0.00	340.97	340.97	0.00	0.00	0.00	340.97	—
Care plan	1 362	1 297	−64.91	1 277	1 274	−3.70	−61.21 (−172.98 to 50.54)	.70
Subtotal			2 047			0.30	2 047	.70
*Direct costs: health care service*								
Nursing home stays	3 023	6 695	3 672	1 334	6 668	5 334	−1 662 (−6 258 to 2 934)	.47
Respite care	98.05	74.71	−23.34	37.94	25.30	−12.64	−10.70 (−243.91 to 215.43	.37
Inpatient hospitalizations	6 548	6 077	−470.94	6 091	8 345	2 254	−2 725 (−6 246 to 795.25)	.12
ED visits	4 829	4 089	−739.76	4 104	6 261	2 157	−2 897 (−5 771 to −23.55)	.08
Outpatient visits	239.96	1 731	1 491	255.08	1 880	1 625	−133.93 (−1 440 to 326.16)	.38
Medications	16 827	10 407	−6 420	18 192	13 240	−4 952	−1 467 (−5 745 to 2 810)	.28
Durable medical equipment	148.97	69.59	−79.38	175.68	79.56	−96.12	16.74 (−104.18 to 137.66)	.96
Visiting nurse	45.87	23.25	−22.62	32.90	28.59	−4.31	−18.31 (−31.94 to 2.50)	.10
Home health aide	411.48	341.74	−67.40	456.37	355.52	−100.85	31.11 (−63.55 to 78.10)	.43
Subtotal			−2 663			6 204	−8 867 (−18 105 to 370.81)	.04
*Direct costs: formal care and social services*								
Social worker	7.79	44.01	36.23	4.03	45.48	41.45	−5.22 (−47.02 to 18.96)	.64
Meals	56.50	36.05	−20.45	39.16	31.40	−7.75	−12.70 (−26.21 to 27.01)	1.00
Transportation	3.36	4.01	0.65	3.63	3.94	0.31	0.34 (−2.28 to 3.80)	.63
Adult day care	280.37	259.11	−21.26	330.19	292.64	−37.55	16.28 (−115.74 to 155.44)	.53
Subtotal			−4.84			−3.55	−1.29 (−215.62 to 97.02)	1.00
*Indirect costs: caregiver time*								
Supervision/assistance with ADLs and IADLs	5 094	3 361	−1 733	5 256	3 368	−1 888	154.32 (−1 210 to 1 391)	.27
*Subtotal*			−1 733			−1 888	154.32 (−1 210 to 1 391)	.27
*Total per-subject mean cost DID*			−2 354			4 313	−6 667 (−15 473 to 2 734)	.27

*Notes*: ADL = activities of daily living; APN = advanced practice nurse; BL = baseline; CI = confidence interval; COPE = Care of Persons with Dementia in their Environments; DID = difference-in-difference; ED = emergency department; IADL = instrumental activities of daily living; M12 = month 12; OT = occupational therapist.

^*a*^Statistical test performed: two-sided Wilcoxon rank-sum test.

### Intervention Costs

COPE intervention costs were $2 047 (mean total per dyad). OT/APN time with the dyad was the costliest component ($677.48), followed by travel time ($402.79), laboratory testing ($340.97), and formal staff training ($218.75).

**Figure 1.**
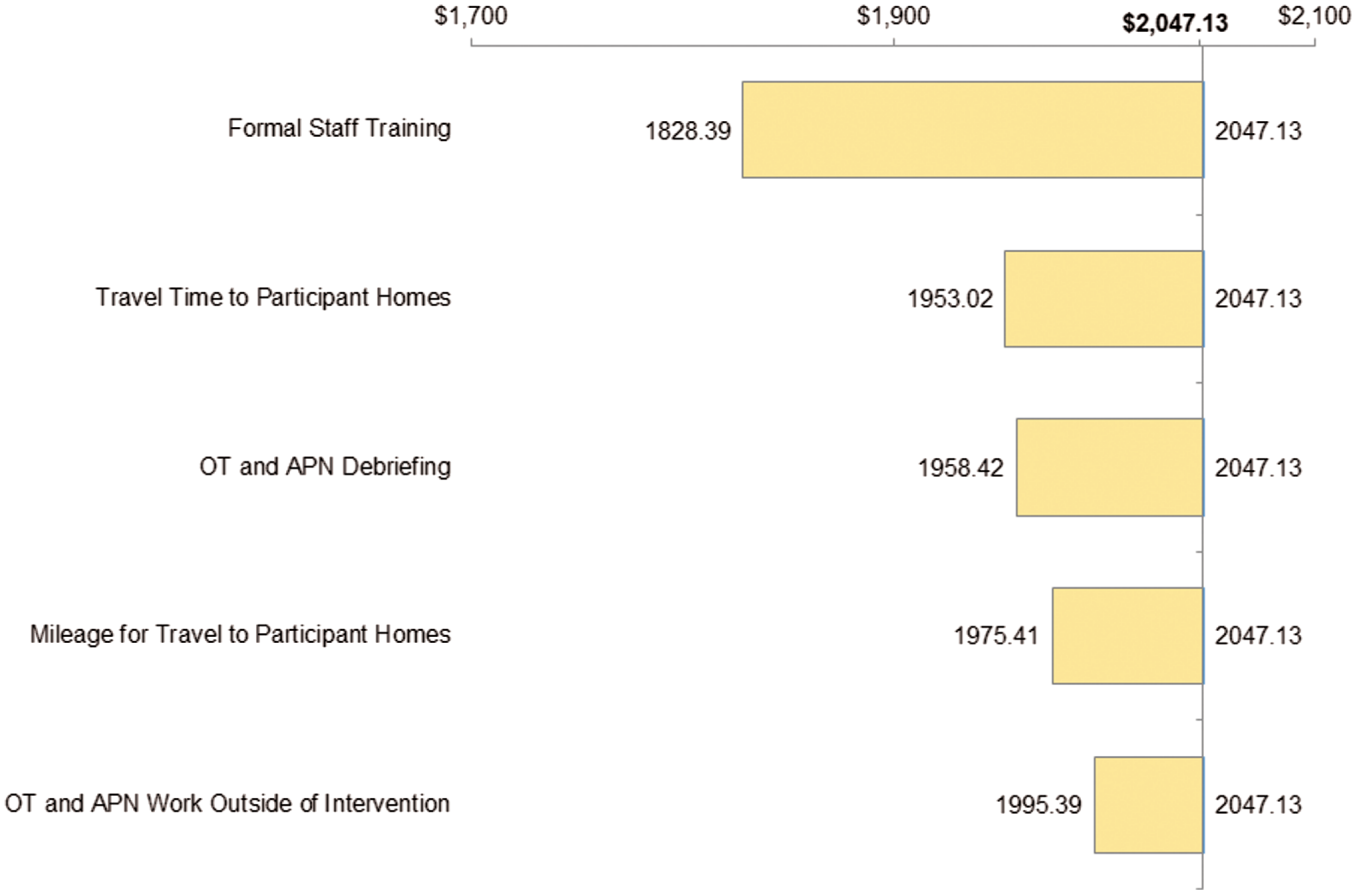
Univariate sensitivity analyses of COPE intervention costs.*Note*: APN = advanced practice nurse; COPE = Care of Persons with Dementia in their Environments; OT = occupational therapist.

Sensitivity analyses on COPE intervention costs (Figure 1) revealed that the following, in order of impact, led to the largest reductions in overall intervention costs: reducing staff training time, OT and APN travel time, OT and APN debriefing, OT and APN mileage for travel to participant’s home, and work outside of COPE delivery. If maximum efficiency is simultaneously achieved for all these categories, COPE’s intervention costs would be $1 522 per dyad.

### Health Care Service Costs

When examining costs for health care services in both cohorts, the COPE group had a mean per-dyad DID of −$8 867 (95% CI: −$18 105, $370.81), representing a cost saving for COPE. These mean per-dyad DID savings were driven by emergency department use (−$739.76 COPE vs $2 157 usual care; net −$2 897 [95% CI: −$5 771, −$23.55]), inpatient hospitalizations (−$470.94 in COPE vs $2 254 in usual care; net −$2 725 [95% CI: −$6 246, $795]), and nursing home stays ($3 672 vs $5 334 in COPE and usual care, respectively; net −$1 662 [95% CI: −$6 258, $2 934]). These differences were not statistically significant.

### Formal Care and Social Services Costs

For costs due to formal care and social services, both cohorts were observed to have savings in HHA costs, meals, and adult day care at 12 months compared to baseline; however, all the DID values were modest and not statistically significant.

### Caregiver Time Costs

For caregiver time, COPE and usual care experienced mean costs of −$1 733 and −$1 888 respectively, yielding a mean per-dyad DID of $154.32 (95% CI: −$1 210, $1 391).

### Assessment of Potential COPE Financing Through the Connecticut HCBS Program

The extent to which COPE would consume CHCPE spending limits is shown in Table 3. The yearly cost for COPE plus home health agency overhead and ongoing CM services is $4 254 regardless of CHCPE category. Considering the annual CHCPE spending limits of $37 716 ($3 143 for 12 months) for Category 2 and $75 432 ($6 286 for 12 months) for Category 3 (13), COPE (plus overhead and ongoing CM) would consume only 11.3% of the annual spending limit for Category 2 and 5.6% of the annual spending limit for Category 3.

**Table 3.** Percent of CHCPE Care Plan Funds Utilized by COPE

CHCPE Category	Care Plan Limits/Month	COPE Cost/Month	COPE + Administration Cost/Month	CM Cost/Month	COPE + Administration Cost/Year + CM/Year	% Consumed by COPE
2	$3 143	$528.01	$739.22	$108.10	$4 254	11.28%
3	$6 286	$528.01	$739.22	$108.10	$4 254	5.64%

*Note*: CHCPE = Connecticut Home Care Program for Elders; COPE = Care of Persons with Dementia in their Environments; CM = care management.

### Assessment of Caregiver’s WTP for COPE in the Context of Connecticut HCBS Program Cost Sharing

At 12 months, caregivers’ median WTP for COPE was $50 per session with 84% of CHCPE Category 2 and 75% of Category 3 caregivers willing to pay at least $25 per session (Supplementary Table A5). These categories have 9% and 0% cost sharing, respectively. For CHCPE Category 2, assuming that the maximum amount spent towards the cost share is 9% of the $3 143 monthly total spending limit ($282.87) and the participant receives a maximum of 3 COPE sessions per month and pays a $25 per session copayment ($75 total), COPE cost sharing would be well below the maximum possible cost sharing borne by participants for the 4 months of the program. For CHCPE Category 3, participants have 0% cost sharing and thus we assumed caregivers would not need to be willing to pay anything for COPE. Therefore, cost sharing for COPE through the Connecticut HCBS program may represent a feasible payment mechanism and would foster sustainability of this intervention.

## Discussion and Implications

The COPE-CT pragmatic trial demonstrated that the COPE intervention can be successfully implemented through Connecticut HCBS. Our CBA revealed that COPE intervention costs also fall within existing CHCPE spending limits, accounting for ongoing CM services to this population, even without consideration of the potential cost savings with COPE. A major strength of our analysis is that we used a CM organization’s data linked to trial data to obtain a robust array of cost measures.

COPE itself costs more than $2 000 per dyad (>$500 per month) above and beyond usual care, but efficiencies in training and delivery could reduce this figure to approximately $1 500. Importantly, however, our CBA suggests potential savings in direct health care costs by COPE participants. Most notably, our findings suggest that COPE participants experienced cost savings in nursing home stays, emergency room use, inpatient hospitalizations, and medications. We postulate that the home support provided by COPE better equipped caregivers to manage dementia, which in turn enabled them to better navigate the care system. Generalized linear models from the main trial analysis indicate improvements in both the person living with dementia’s behavioral and psychological symptoms score as well as the caregiver’s own perceived well-being (11).

As recommended by the 2018 National Research Summit on Care, Services, and Supports for Persons with Dementia and Their Caregivers, efficacious programs that demonstrate positive outcomes for persons living with dementia and their caregivers also need viable payment models to support scaling, dissemination, and sustainability (6). In this study, we tested embedding the COPE program into Connecticut HCBS. Considering that COPE only utilizes 5.6%–11.3% of the yearly CHCPE care plan limits, COPE could likely be covered through this program. Though HCBS programs vary from state to state in terms of eligibility, spending limits, and covered services, our findings from Connecticut provide a proof-of-concept that warrants consideration by other states.

Another option for a payment model would be through Medicare. Boustani et al. (39) have recently recommended that the Centers for Medicare and Medicaid Services provide a per beneficiary per month payment to cover comprehensive dementia care using collaborative dementia care models for the person living with dementia and support and education for their unpaid caregivers. The Alzheimer’s Association and the Alzheimer’s Impact Movement also published a Medicare-based alternate payment model proposal, with key elements including a capitated and performance based payment structure (40). Overall, some key conditions for sustainable payment for COPE under Medicare would require that visits by the OT and APN be covered in type and number, specific billing codes be established for the services, costs for COPE to not exceed benefit and or reimbursement caps, the 12 COPE visits (10 OT, 2 APN) be recognized as a “package” to avoid rejections and interruptions, and for COPE to be authorized by appropriate personnel. Of note, the new Medicare ADRD care planning service codes are not sufficient for the COPE program (41). As these payment approaches evolve, it is becoming apparent that there will not be a single payment model suitable for all dementia nonpharmaceutical interventions.

There are very few published economic analyses examining dementia care programs that support both persons living with dementia and caregivers. In a study assessing the cost–benefit of 10 sessions of OT at home over 5 weeks versus usual care in persons living with dementia and caregiver dyads, the intervention group achieved a cost savings of €1 748 ($2 737, 2019 USD) in total care costs, though this was nonsignificant (42). This difference was driven by reduction of informal care costs (care given by offspring, neighbors), nursing home costs, and hospitalization costs (42). In another pilot program that examined the impact of a care coordination and support partnership between a telephonic nursing program and a home care organization in Alzheimer’s patients and caregiver dyads, the intervention group had lower average inpatient costs compared to the historical control group, at $12 989 and $30 650, respectively (*p* < .011) (43). However, the use of a historical control group makes it difficult to discern the actual impact of the intervention. With respect to WTP, a study assessing caregiver WTP for a similar in-home intervention to help manage behavioral symptoms and caregiver stress indicated that the mean adjusted WTP at baseline was $36 per session (44), which is similar to WTP values for COPE.

The study has several limitations. First, the person living with dementia in this study may have been more advanced in their disease than in other populations of community-dwelling persons living with dementia, as evidenced by the increase in nursing home costs for both groups over the course of their 12 months in the study. While this study cohort may not be representative of all community-dwelling persons living with dementia, they might be more representative of persons living with dementia enrolled in Medicaid waiver and state-funded HCBS programs in other states that target those at risk or eligible for nursing home entry. Second, the study was powered on the main outcomes for the parent trial but not on the cost study, and we were unable to detect statistical significance between the groups. This is common for cost assessments conducted alongside clinical trials (45). Third, information regarding health care resource utilization was obtained either from the CCC database (informed through biannual interviews of the caregiver by the CCC care manager) or through study interviews, as claims data were not available for this analysis. Fourth, the WTP question only asked about willingness to pay, without considering ability to pay. Thus, the WTP reported does not necessarily equate to a financial ability to pay; however, it should be noted that the per-session WTP reported are modest and likely achievable for many dyads. Finally, we assessed possible financing of COPE through Connecticut HCBS only, but the concept may be replicable in other states’ HCBS programs depending on their eligibility criteria and spending thresholds. Further research is needed to understand how COPE may be afforded through other state HCBS programs.

The COPE program trended toward net cost savings in several direct health care cost categories including emergency department visits, inpatient hospitalizations, and medications, and can be afforded by the Connecticut CHCPE based on current monthly cost caps and cost-sharing percentages. State- and Medicaid-funded HCBS programs represent an important, sustainable payment model for delivering nonpharmacological dementia interventions such as COPE.

## Supplementary Material

igab042_suppl_Supplementary_Materials
