# Supplementary material for: Cost–Benefit Analysis of the COPE Program for Persons Living With Dementia: Toward a Payment Model
Source: Innov Aging. 2021 Oct 16;6(1):igab042. doi: 10.1093/geroni/igab042 (PMC8763605; doi:10.1093/geroni/igab042)
Supplement: igab042_suppl_Supplementary_Materials [file igab042_suppl_supplementary_materials.docx]

**Online Supplementary Material**

Figure A1. Consort chart

*Notes.* CCC = Connecticut Community Care

Table A1. Comparison of Demographics of Caregiver and Persons Living with Dementia Dyads in the Cost Sample Group to Non-Cost Sample Group

| **Characteristic** | **COPE CS**  N = 130^a^ | **COPE NCS**  N = 15^a^ | **p-value**^b^ | **UC CS**  N = 120^a^ | **UC NCS**  N = 26^a^ | **p-value**^b^ |
| --- | --- | --- | --- | --- | --- | --- |
| CHCPE Category |  |  | 0.6427 |  |  | 0.1104 |
| 1 | 2 (1.5%) | 0 (0.0%) |  | 1 (0.8%) | 1 (3.8%) |  |
| 2 | 38 (29.2%) | 3 (20.0%) |  | 37 (30.8%) | 4 (15.4%) |  |
| 3 | 90 (69.2%) | 12 (80.0%) |  | 82 (68.3%) | 21 (80.8%) |  |
| Caregiver Age | 62.1 (11.25) | 58.4 (14.25) | 0.3476 | 63.0 (10.58) | 61.0 (12.79) | 0.4531 |
| Person Living with Dementia Age | 85.1 (8.31) | 84.5 (9.48) | 0.8163 | 85.4 (7.50) | 82.7 (7.72) | 0.1169 |
| Caregiver Gender |  |  | **0.0097** |  |  | 1 |
| Female | 103 (79.2%) | 7 (46.7%) |  | 76 (63.3%) | 16 (61.5%) |  |
| Male | 27 (20.8%) | 8 (53.3%) |  | 44 (36.7%) | 10 (38.5%) |  |
| Person Living with Dementia Gender | |  | 0.1186 |  |  | 1 |
| Female | 96 (73.8%) | 14 (93.3%) |  | 94 (78.3%) | 21 (80.8%) |  |
| Male | 34 (26.2%) | 1 (6.7%) |  | 26 (21.7%) | 5 (19.2%) |  |
| Caregiver Race |  |  | 0.0858 |  |  | 0.2263 |
| White, Caucasian | 95 (73.1%) | 10 (66.7%) |  | 94 (78.3%) | 22 (84.6%) |  |
| Black, African-American | 27 (20.8%) | 2 (13.3%) |  | 19 (15.8%) | 1 (3.8%) |  |
| Other | 6 (4.6%) | 2 (13.3%) |  | 6 (5.0%) | 3 (11.5%) |  |
| Unknown / No response | 2 (1.5%) | 0 (0.0%) |  | 0 (0.0%) | 0 (0.0%) |  |
| Native American or Alaska native | 0 (0.0%) | 1 (6.7%) |  | 1 (0.8%) | 0 (0.0%) |  |
| Person Living with Dementia Race |  |  | 0.3626 |  |  | 0.3923 |
| White, Caucasian | 98 (75.4%) | 10 (66.7%) |  | 94 (78.3%) | 24 (92.3%) |  |
| Black, African-American | 25 (19.2%) | 3 (20.0%) |  | 19 (15.8%) | 1 (3.8%) |  |
| Other | 7 (5.4%) | 2 (13.3%) |  | 6 (5.0%) | 1 (3.8%) |  |
| Native American or Alaska native | 0 (0.0%) | 0 (0.0%) |  | 1 (0.8%) | 0 (0.0%) |  |
| Caregiver Highest Education Attained | |  | 0.4212 |  |  | 0.4581 |
| College / Postgrad | 66 (50.8%) | 5 (33.3%) |  | 50 (41.7%) | 8 (30.8%) |  |
| Some college | 32 (24.6%) | 5 (33.3%) |  | 29 (24.2%) | 9 (34.6%) |  |
| HS or less | 31 (23.8%) | 5 (33.3%) |  | 41 (34.2%) | 9 (34.6%) |  |
| Unknown / No response | 1 (0.8%) | 0 (0.0%) |  | 0 (0.0%) | 0 (0.0%) |  |
| Person Living with Dementia Highest Education Attained | | | 0.2064 |  |  | 0.1357 |
| HS graduate | 73 (56.2%) | 10 (66.7%) |  | 66 (55.0%) | 9 (34.6%) |  |
| Less than HS | 34 (26.2%) | 5 (33.3%) |  | 37 (30.8%) | 11 (42.3%) |  |
| College / Postgrad | 23 (17.7%) | 0 (0.0%) |  | 17 (14.2%) | 6 (23.1%) |  |
| Caregiver Employment Status |  |  | 0.8019 |  |  | 1 |
| Not working | 64 (49.2%) | 9 (60.0%) |  | 56 (46.7%) | 13 (50.0%) |  |
| Full time (> 35 hrs/wk) | 48 (36.9%) | 5 (33.3%) |  | 51 (42.5%) | 11 (42.3%) |  |
| Part time (< 35 hrs/wk) | 18 (13.8%) | 1 (6.7%) |  | 13 (10.8%) | 2 (7.7%) |  |
| Caregiver Difficulty Paying for the Basics | |  | 0.9271 |  |  | 0.5312 |
| Not difficult at all | 67 (51.5%) | 9 (60.0%) |  | 49 (40.8%) | 13 (50.0%) |  |
| Somewhat difficult | 33 (25.4%) | 4 (26.7%) |  | 34 (28.3%) | 9 (34.6%) |  |
| Not very difficult | 20 (15.4%) | 1 (6.7%) |  | 25 (20.8%) | 3 (11.5%) |  |
| Very difficult | 9 (6.9%) | 1 (6.7%) |  | 12 (10.0%) | 1 (3.8%) |  |
| Unknown / No response | 1 (0.8%) | 0 (0.0%) |  | 0 (0.0%) | 0 (0.0%) |  |
| Caregiver Marital Status |  |  | 0.9669 |  |  | 0.6127 |
| Married or living as married | 74 (56.9%) | 10 (66.7%) |  | 73 (60.8%) | 14 (53.8%) |  |
| Divorced/Separated | 24 (18.5%) | 3 (20.0%) |  | 20 (16.7%) | 5 (19.2%) |  |
| Never married | 25 (19.2%) | 2 (13.3%) |  | 17 (14.2%) | 6 (23.1%) |  |
| Widowed, not currently married | 7 (5.4%) | 0 (0.0%) |  | 10 (8.3%) | 1 (3.8%) |  |
| Caregiver Relationship to Person Living with Dementia | | | **0.0356** |  |  | 0.9476 |
| Daughter | 78 (60.0%) | 6 (40.0%) |  | 62 (51.7%) | 13 (50.0%) |  |
| Spouse | 14 (10.8%) | 6 (40.0%) |  | 26 (21.7%) | 5 (19.2%) |  |
| Son | 23 (17.7%) | 2 (13.3%) |  | 21 (17.5%) | 6 (23.1%) |  |
| Other | 15 (11.5%) | 1 (6.7%) |  | 11 (9.2%) | 2 (7.7%) |  |
| Caregiver and Person Living with Dementia Living Arrangement | | | 1 |  |  | 0.0763 |
| Live together | 75 (57.7%) | 9 (60.0%) |  | 68 (56.7%) | 20 (76.9%) |  |
| Live apart | 55 (42.3%) | 6 (40.0%) |  | 52 (43.3%) | 6 (23.1%) |  |

*Notes.* CS = cost sample; NCS = not in cost sample; UC = usual care.

^a^ Statistics presented: mean (SD); n (%); ^b^ Statistical tests performed: t-test; Fisher's exact test

Table A2. Data Source of Variables

| **Variable** | **Data Source** | **Pertains to (Dyad, caregiver, or person living with dementia)** |
| --- | --- | --- |
| **Direct Costs: Delivery of Intervention or Usual Care Control** | | |
| Formal Staff Training | Study logs | N/A |
| Screening for Program Eligibility | Assumption | Dyad |
| Intervention Delivery | Study logs | Dyad |
| OT/APN Work Outside of Intervention | Study logs | Dyad |
| Travel Time to Participant Homes | Study logs | Dyad |
| Mileage for Travel to Participant Homes | Study logs | Dyad |
| Interventionist Debriefing | Study logs | Dyad |
| Activity Supplies and Assessment Materials | Study expense logs | Dyad |
| Laboratory Testing | Study expense logs | Person living with dementia |
| Monthly Care Plan | CCC | Person living with dementia |
| **Direct costs: healthcare utilization** | | |
| Nursing Home Admissions | CCC | Person living with dementia |
| Respite Care | Study interviews | Person living with dementia |
| Inpatient Hospitalizations | CCC | Person living with dementia |
| ED Visits | CCC | Person living with dementia |
| Outpatient Visits | Study interviews | Person living with dementia |
| Medications | CCC | Person living with dementia |
| Durable Medical Equipment | CCC | Person living with dementia |
| Visiting Nurse | Study interviews | Person living with dementia |
| Home Health Aide | Study interviews | Person living with dementia |
| **Direct Costs: Formal Care and Social Services** | | |
| Social Worker | Study interviews | Person living with dementia |
| Meals | Study interviews | Person living with dementia |
| Transportation | Study interviews | Person living with dementia |
| Adult Day Care | Study interviews | Person living with dementia |
| **Indirect Costs: Caregiver Time** | | |
| Supervision/Assistance with ADLs and IADLs | Study interviews | Caregiver |

*Notes*. OT = Occupational Therapist; APN = Advanced Practice Nurse; CCC = Connecticut Community Care; ED = Emergency Department; ADL = Activities of Daily Living; IADL = Instrumental Activities of Daily Living.

^a^ Statistical test performed: Two-sided Wilcoxon rank-sum test.

Table A3. Cost assumptions for outpatient visits by provider type

| **Provider Type** | **CPT Code** | **Cost ($US 2019)^a^** |
| --- | --- | --- |
| Primary care physician | 99214 | $183.18 |
| Geriatrician or neurologist | 99243 | $297.05 |
| Psychiatrist | 90791 | $215.06 |
| Psychologist | 99203 | $199.10 |
| Physical therapist | 97001 | $158.51 |
| Occupational therapist or speech language pathologist | 97003 | $158.51 |

*Notes.* ^a^ The costs in this table reflect the values in the 2015 National Fee Analyzer inflated to $US 2019, consistent with the year of valuation for this analysis.

Figure A2. COPE Financing Through HCBS: A Conceptual Illustration of a Payment Model


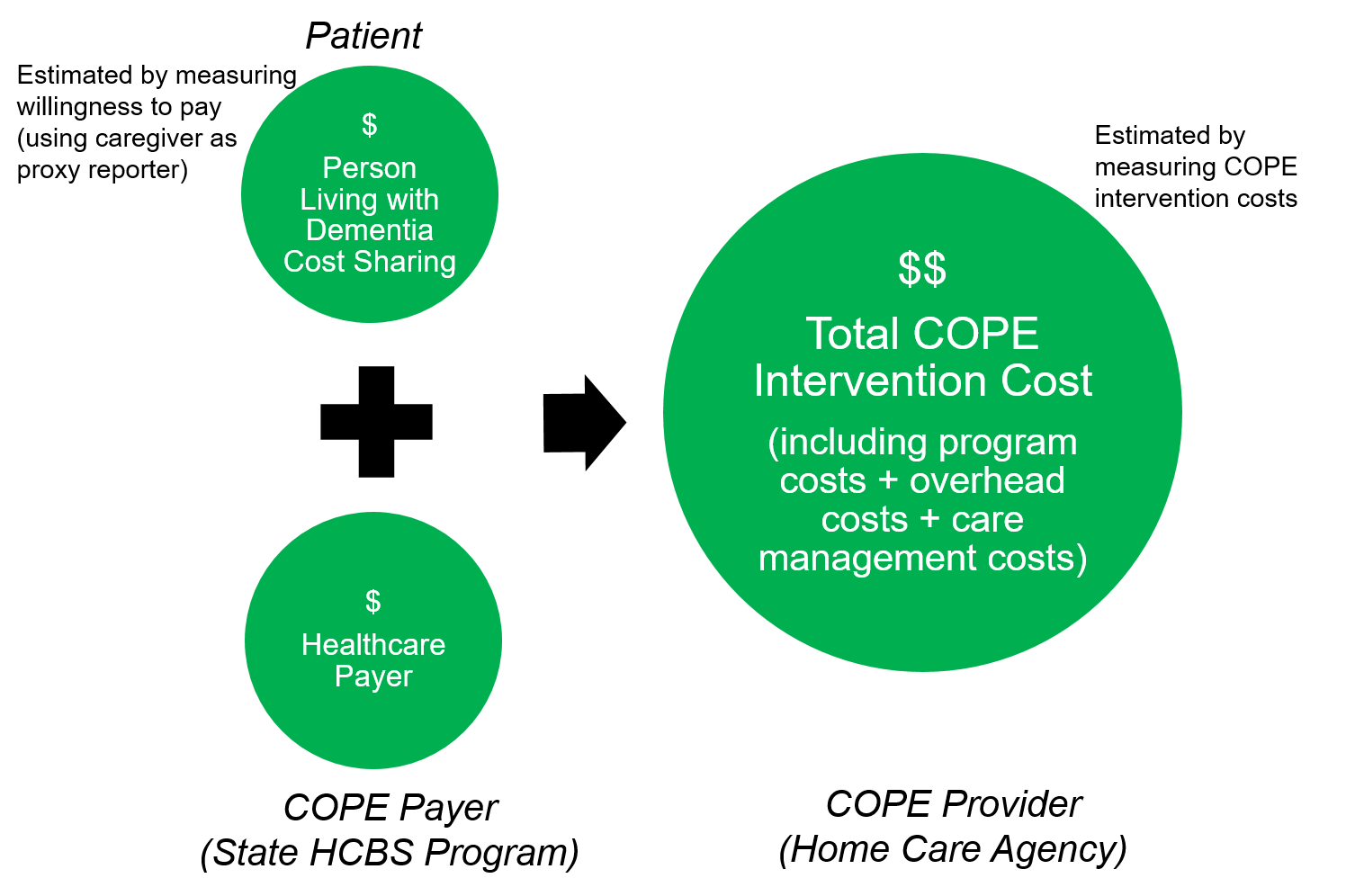


Table A4. Healthcare Utilization at Baseline and Month 12

|  | **COPE Intervention (n=130)** | | | | | | | **Usual Care Control (n=120)** | | | | | | |  |  |
| --- | --- | --- | --- | --- | --- | --- | --- | --- | --- | --- | --- | --- | --- | --- | --- | --- |
|  | **Total** | **Mean** | **SD** | **Total** | **Mean** | **SD** | **M12-BL MEAN DIFF** | **Total** | **Mean** | **SD** | **Total** | **Mean** | **SD** | **M12-BL MEAN DIFF** | **MEAN DID (95% CI)** | **p-value^a^** |
| **Healthcare Utilization** | | | | | | | | | | | | | | | | |
| Nursing Home Stays | 35 | 0.27 | 0.63 | 58 | 0.45 | 0.90 | 0.18 | 22 | 0.18 | 0.52 | 53 | 0.44 | 0.82 | 0.26 | -0.08 (-0.328, 0.166) | 0.279 |
| Respite Care | 84 | 0.65 | 4.21 | 64 | 0.49 | 3.78 | -0.15 | 30 | 0.25 | 2.74 | 20 | 0.17 | 1.12 | -0.08 | -0.07  (-1.607, 1.420) | 0.370 |
| Inpatient Hospitalizations | 59 | 0.45 | 0.84 | 67 | 0.52 | 0.83 | 0.06 | 46 | 0.38 | 0.66 | 76 | 0.63 | 0.89 | 0.25 | -0.19  (-0.420, 0.053) | 0.167 |
| ED Visits | 47 | 0.36 | 0.81 | 40 | 0.31 | 0.70 | -0.05 | 38 | 0.32 | 0.74 | 56 | 0.47 | 0.72 | 0.15 | -0.2  (-0.41, 0.003) | 0.027 |
| Outpatient Visits | 167 | 1.28 | 2.43 | 1,299 | 9.99 | 19.35 | 8.71 | 169 | 1.41 | 3.00 | 1,283 | 10.69 | 18.17 | 9.28 | -0.58  (-8.330, 2.286) | 0.484 |
| Medications | 1,327 | 10.21 | 7.65 | 954 | 7.34 | 5.76 | -2.87 | 1,108 | 9.23 | 5.67 | 837 | 6.98 | 5.00 | -2.26 | -0.61  (-2.300, 1.074) | 0.348 |
| Durable Medical Equipment | 200 | 1.54 | 2.12 | 97 | 0.75 | 1.68 | -0.79 | 183 | 1.53 | 2.47 | 79 | 0.66 | 1.19 | -0.87 | 0.07  (-0.625, 0.773) | 0.992 |
| Visiting Nurse | 219 | 1.68 | 2.57 | 111 | 0.85 | 1.73 | -0.83 | 145 | 1.21 | 1.99 | 126 | 1.05 | 2.01 | -0.16 | -0.67  (-1.173, 0.092) | 0.108 |
| Home Health Aide | 2,820 | 21.69 | 12.31 | 2,342 | 18.02 | 13.61 | -3.68 | 2,887 | 24.06 | 14.72 | 2,249 | 18.74 | 16.54 | -5.32 | 1.64  (-3.351, 4.117) | 0.438 |
| **Formal Care and Social Services** | | | | | | | | | | | | | | | | |
| Social Worker | 23 | 0.18 | 0.44 | 130 | 1.00 | 2.16 | 0.82 | 11 | 0.09 | 0.29 | 124 | 1.03 | 2.38 | 0.94 | -0.12  (-1.068, 0.431) | 0.636 |
| Meals | 616 | 4.74 | 9.29 | 393 | 3.02 | 7.42 | -1.72 | 394 | 3.28 | 8.03 | 316 | 2.63 | 6.85 | -0.65 | -1.07  (-2.198, 2.265) | 0.991 |
| Transportation | 270 | 2.08 | 6.08 | 322 | 2.48 | 7.12 | 0.40 | 269 | 2.24 | 6.21 | 292 | 2.43 | 6.93 | 0.19 | 0.21  (-1.412, 2.351) | 0.634 |
| Adult Day Care | 356 | 2.74 | 6.46 | 329 | 2.53 | 6.27 | -0.21 | 387 | 3.23 | 7.47 | 343 | 2.86 | 6.68 | -0.37 | 0.16  (-1.131, 1.518) | 0.526 |

*Notes.* BL = baseline; M12 = month twelve; DID = difference-in-difference; CI = confidence interval; OT = occupational therapist; APN = advanced practice nurse; ED = emergency department; ADL = activities of daily living; IADL = instrumental activities of

^a^ Statistical test performed: Two-sided Wilcoxon rank-sum test.

Table A5. WTP among dyads receiving COPE

| **CHCPE Category** | **Timepoint** | **N** | **%WTP $20^a^** | **%WTP $25^a^** | **%WTP $50^a^** |
| --- | --- | --- | --- | --- | --- |
| 2 | BL | 37 | 73% | 73% | 43% |
| 2 | M12 | 31 | 84% | 84% | 65% |
| 3 | BL | 90 | 74% | 74% | 46% |
| 3 | M12 | 79 | 77% | 75% | 54% |

*Notes.* N = number of dyads reporting WTP; BL = baseline. M12 = month twelve.

^a^ WTP was reported by caregivers. Caregivers were WTP at least this amount.
